# Mechanisms of resistance to immune checkpoint inhibitors in renal cell carcinoma and novel treatment strategies

**DOI:** 10.3389/fonc.2026.1984645

**Published:** 2026-10-19

**Authors:** Li Chen, Jie Cheng, Shilong Mao

**Affiliations:** 1 Department of Pharmacy, Shanghai Xuhui Central Hospital, Zhongshan-Xuhui Hospital, Fudan University, Shanghai, China; 2 Department of Urology, Shanghai Xuhui Central Hospital, Zhongshan-Xuhui Hospital, Fudan University, Shanghai, China

**Keywords:** combination therapy, immune checkpoint inhibitor, immunotherapy resistance, renal cell carcinoma, tumor microenvironment

## Abstract

In recent years, immune checkpoint inhibitors (ICIs), such as nivolumab, ipilimumab, pembrolizumab, and atezolizumab have substantially improved the prognosis of patients with advanced renal cell carcinoma. These agents have greatly changed the management of advanced renal cell carcinoma, shifting the treatment paradigm from cytokine and vascular endothelial growth factor-based therapies to immunotherapy strategies. However, drug resistance limits the long-term benefit. The mechanisms of immunotherapy resistance in RCC are complex. Recently, increasing research has focused on how cancer cells evade the surveillance and attack of the immune system and identifying the molecular and cellular drivers of resistance. In response, combination regimens incorporating ICIs are being developed to alleviate drug resistance and attack cancer cells through multiple pathways. At the same time, novel targeted strategies are under investigation to develop innovative approaches to delay, partially overcome, or bypass resistance in defined clinical settings. This review outlines the mechanisms of immunotherapy resistance in RCC from three interrelated perspectives: cancer cell-intrinsic mechanisms, the tumor microenvironment, and host related factors. We also summarize current efforts to overcome resistance, including ICI combined with tyrosine kinase inhibitors, dual ICI therapy, tumor microenvironment-targeting strategies, radiotherapy combined with immunotherapy, and gut microbiota modulation. Although these new strategies have shown different therapeutic effects in clinical trials, deeper understanding of immune regulation highlights their potential. Moreover, the integration of multi-omics analysis, and microbiome regulation may increase patient choices and optimize therapeutic outcomes, but their clinical efficacy has not been fully confirmed and requires prospective, treatment defined validation. Overall, ICIs have substantially changed RCC treatment, future progress will depend on deeper biological insights, rational combination design, and validated personalized immunotherapy.

## 1 Introduction

Renal cell carcinoma (RCC) is a malignant tumor originating from renal tubular epithelium cells and is one of the most common renal malignant tumor (1). Its incidence continues to rise globally. Approximately 30% of patients present with metastasis at initial diagnosis (1). Among advanced cases, postoperative recurrence occurs in 30-40%, and 50% develop distant metastasis with a poor prognosis (1). Clear cell renal cell carcinoma (ccRCC) is the most common subtype, accounting for approximately 70% of cases, and is characterized by tumor cells with clear or lightly stained cytoplasm (2). The main pathogenic mechanism of ccRCC involves inactivation of the von Hippel Lindau (VHL) gene, leading to abnormal stability and accumulation of hypoxia-inducible factor 2α (HIF-2α), thereby promoting tumor growth. This in turn drives overexpression of angiogenic factors, including vascular endothelial growth factor (VEGF) and platelet-derived growth factor (PDGF), providing a strong biological basis for anti-angiogenic therapy. The high immunogenicity and T-cell infiltration of RCC, in turn, provide the rationale for immunotherapy (2).

RCC has historically been regarded as relatively resistant to conventional radiotherapy and chemotherapy (1). In the targeted therapy era, tyrosine kinase inhibitors (TKIs) significantly extended progression-free survival (PFS) in advanced RCC. Nevertheless, objective response rates remain modest, and acquired resistance is nearly inevitable (2).

The emergence of ICIs targeting programmed cell death protein-1 (PD-1), programmed cell death ligand 1 (PD-L1), and cytotoxic T-lymphocyte-associated protein 4 (CTLA-4), has revolutionized RCC treatment. Randomized trials have shown regimen- and endpoint-specific benefits. Nivolumab plus ipilimumab improved OS versus sunitinib in CheckMate 214 (3, 4); pembrolizumab plus axitinib and nivolumab plus cabozantinib improved PFS and OS versus sunitinib (5, 6); and lenvatinib plus pembrolizumab improved PFS and OS versus sunitinib (7). Currently, ICI-TKI combinations, such as pembrolizumab plus axitinib, nivolumab plus cabozantinib, as well as dual ICI therapy with nivolumab plus ipilimumab, are standard first-line treatments recommended by international guidelines. These ICI-based combinations significantly improve the survival in advanced RCC patients (8). However, the IMmotion151 analysis did not demonstrate an OS advantage for atezolizumab plus bevacizumab over sunitinib (9). Thus, ICI-based regimens should not be presented as conferring uniform survival benefit. Immunotherapy is not universally effective either. Some patients do not respond initially, and even initial responders often lose benefit, indicating primary and acquired resistance (8). The mechanisms of RCC resistance to ICIs remain poorly understood. Unlike most immunotherapy sensitive tumors, RCC displays unique immunological features. It exhibits high immunogenicity with abundant T-cell infiltration in the tumor microenvironment (TME), providing a robust biological foundation for ICIs (1). Paradoxically, high T-cell infiltration is not consistently associated with improved ICI outcomes in RCC (8). This contradictory phenotype suggests that resistance mechanisms in ccRCC differ substantially from those in other tumors (8). In addition, the tumor mutational burden (TMB) of RCC is relatively low among solid tumors, making ICI efficacy difficult to predict (8). The predictive value of traditional biomarkers such as TMB and PD-L1 expression is limited, highlighting the urgency of exploring RCC immune resistance mechanism in depth.

Recent progress in single-cell sequencing and spatial transcriptomics has deepened our understanding of resistance in renal cancer. Enrichment of immunosuppressive myeloid cells, T-cell exhaustion, lack of B cells and tertiary lymphoid structure, and recruitment of regulatory T cells (Tregs) in the TME are all considered as potential drivers of resistance (8). In parallel, treatment strategies have evolved from simple drug superposition to mechanism-driven combination and sequencing. The related clinical trials of HIF-2α inhibitor Belzutifan (10, 73), retrospective studies of stereotactic radiotherapy combined with systemic therapy (11), and emergence of novel immune targets such as lymphocyte activation gene-3 (LAG-3) and T cell immunoreceptor with Ig and ITIM domains (TIGIT) offer new avenues for overcoming resistance (12). Novel biomarker discovery and rational combination strategies have thus become key frontier directions.

However, existing reviews on RCC immunotherapy resistance have largely organized the field by mechanism category, enumerating intrinsic, microenvironmental, and host-related factors without systematically assessing which mechanisms are supported by sufficient evidence to serve as intervention targets. This review does not aim to provide another comprehensive catalog of resistance mechanisms. Instead, we propose a mechanism-to-intervention synthesis diagram that grades each resistance mechanism by evidence strength (strong, moderate, weak), direction of effect (resistance-conferring, sensitivity-conferring, or context-dependent), resistance type (primary vs acquired), and druggability (clinically actionable, preclinical, or hypothesis only). The distinct contribution of this review is therefore to clarify which findings genuinely change the interpretation of resistance and which therapeutic approaches should be prioritized, rather than merely restating the complexity of the resistance network.

This article provides a narrative review of the multi-level mechanisms of immunotherapy resistance in RCC and of integrated strategies to overcome it, intended as a balanced overview rather than a systematic synthesis. References were identified through PubMed. The final search was performed on September 26, 2026. The main search concepts combined terms related to RCC, including RCC, ccRCC, ICIs, and tumor-intrinsic, microenvironmental, and host-related resistance mechanisms, with priority given to randomized trials, large cohorts, direct RCC/ccRCC mechanistic studies, and negative or conflicting results. The scope is RCC, predominantly ccRCC, and extrapolations beyond ccRCC or the advanced setting are flagged. As a narrative review, no protocol registration, PRISMA reporting, meta-analysis, or new experiments were undertaken.

## 2 Mechanisms of resistance to ICIs in RCC

Drug resistance in RCC is multifactorial, involving tumor-intrinsic alterations and extrinsic immune escape mechanisms, such as an immunosuppressive microenvironment and host-related factors. These factors create an unfavorable environment, weakening effector T-cell transport, survival, and function, and leading to immunotherapy resistance (**Figure 1**).

**Figure 1:**
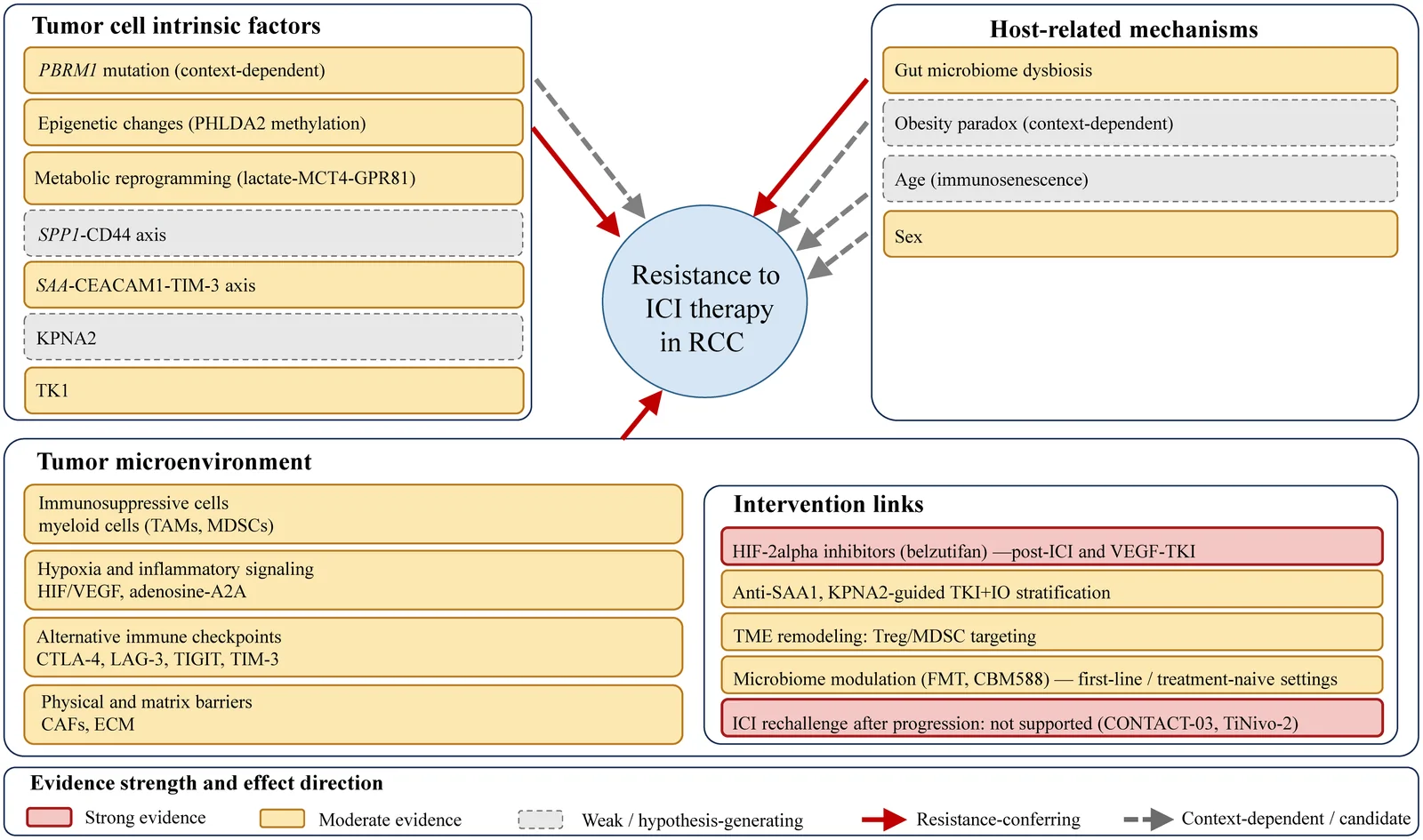
Multilevel mechanisms of immunotherapy resistance in renal cell carcinoma (RCC) and candidate intervention links. Resistance mechanisms are organized into three interrelated compartments: tumor cell-intrinsic factors, the tumor microenvironment (TME), and host-related mechanisms. Box color denotes evidence strength: red, strong evidence; yellow, moderate evidence; gray dashed, weak evidence. Arrow style denotes effect direction: red solid arrow, resistance-conferring; gray dashed arrow, context-dependent. Box color and arrow style are graded independently. For mechanisms with context-dependent evidence (e.g., obesity paradox, *PBRM1* mutation), the direction of effect is specified in the corresponding section of the main text. Evidence strength was graded as strong (supported by randomized trials, or by consistent direct RCC/ccRCC clinical-cohort and functional evidence), moderate (supported by large retrospective cohorts, single-arm trials, or reproducible mechanistic studies with partial clinical validation), and weak (associational, preclinical, or conflicting evidence requiring prospective validation). Belzutifan is indicated for post-ICI and VEGF-TKI settings in advanced ccRCC. ICI, immune checkpoint inhibitor; TME, tumor microenvironment; TAM, tumor-associated macrophage; MDSC, myeloid-derived suppressor cell; NET, neutrophil extracellular trap; Treg, regulatory T cell; CAF, cancer-associated fibroblast; ECM, extracellular matrix; FMT, fecal microbiota transplantation; CBM588, *Clostridium butyricum*.

### 2.1 Tumor cell intrinsic factors

Renal cancer cells acquire the ability to resist immune attacks through key gene mutations, activation of signaling pathways, metabolic changes, and other mechanisms.

#### 2.1.1 PBRM1 gene mutation

polybromo 1 (PBRM1) is a subunit of the PBAF (SWI/SNF) chromatin remodeling complex. It is the second most common mutated gene in RCC, with approximately 40% of cases harboring loss-of-function mutations. Clinical evidence regarding PBRM1 in immunotherapy resistance remains controversial.

Several studies from CRISPR-Cas9 screening to patient-cohort analyses suggest that loss of PBRM1 function reduces resistance to ICI therapy, with PBRM1 inactivated patients showing higher response rate to PD-1/PD-L1 inhibitors (13, 14). *Miao* et al. analyzed 35 patients with renal cancer treated with PD-1 inhibitor and found no significant differences in PD-L1 expression, drug dosage or tumor size between responders and non-responders (14). In the 35-patient discovery cohort, PBRM1 mutations were significantly enriched in patients with clinical benefit versus those without (82% vs 23%) (14). Given the very small sample size and the exploratory, single-cohort discovery design of this analysis, these estimates are imprecise and should not be regarded as a validated effect size. Mechanistically, PBRM1 mutations alter transcriptional output in Janus kinase-signal transducers and activators of transcription (JAK-STAT), hypoxia and immune signaling pathways (14). This early positive signal has not been consistently reproduced in larger, adequately powered cohorts.

Conversely, subsequent clinical and mechanistic studies, including analyses with independent cohort validation and murine models, reached the opposite conclusion. *Liu* et al., through retrospective analysis of the IMmotion150 RCC study, confirmed that PBRM1 loss is associated with lower TME immunogenicity and upregulated of angiogenesis, and found that PBRM1 mutation reduced ICI benefit (15). PBRM1 loss defined a non-immunogenic tumor phenotype associated with ICI resistance (15). In mouse models, PBRM1-deficient subcutaneous renal tumors showed stronger ICI resistance (15). PBRM1 mutation mediated immunotherapy resistance by inhibiting IFNγ-STAT1 signaling and promoting an angiogenesis phenotype (15). Recent studies further showed that PBRM1 deficiency reshapes the immunosuppressive microenvironment through epigenetic regulation of the PBRM1-KDM5C-IL6 axis (16).

Importantly, the apparent contradiction is more likely to reflect differences in study design, patient composition, treatment line, prior VEGF exposure, definition of PBRM1 loss, and endpoints. Miao et al. analyzed 35 patients treated with PD-1 monotherapy, predominantly in the later-line setting, defined PBRM1 alteration by exome sequencing-detected loss-of-function mutations, and used objective response rate as the primary endpoint (14). In contrast, Liu et al. analyzed the IMmotion150 cohort, which included both treatment-naïve and pretreated patients, used a combined definition of PBRM1 protein loss and mutation, assessed PFS and OS, and incorporated murine validation (15). The two studies differ substantially in prior VEGF exposure, treatment regimen, definition of PBRM1 loss, endpoint selection, and model systems. More recent evidence supports a context-dependent interpretation. In a multi-cohort analysis, concurrent PBRM1 and RAD51 deficiency identified a subgroup with exceptional sensitivity to immune checkpoint blockade, characterized by M1 macrophage enrichment, contrasting with the non-immunogenic phenotype associated with isolated PBRM1 loss (17). In addition, PBRM1 alteration and PD-L1 positivity frequently co-occur (approximately 60% of PBRM1-altered cases were PD-L1 positive), although the association did not reach statistical significance (18). These observations suggest that the “PBRM1 paradox” largely reflects distinct immune phenotypes determined by treatment context, co-mutation status, such as RAD51, and the definition of PBRM1 loss, rather than a uniform resistance- or sensitivity-conferring biomarker. This has direct implications for intervention priorities: strategies targeting PBRM1-deficient tumors should not aim to restore PBRM1 itself, but should prioritize downstream actionable nodes, including IFNγ-STAT1 signaling, hypoxia/angiogenesis, and the IL-6 axis, with clinical trials stratified by treatment line and prior VEGF exposure.

Collectively, the evidence does not support a simple binary classification of PBRM1 as either resistance- or sensitivity-conferring. The early fourfold response-rate difference was derived from a 35-patient discovery cohort and shouldnot be viewed as a confirmed biomarker effect, particularly because subsequent larger clinical and mechanistic studies have predominantly associated PBRM1 loss with an immunosuppressive phenotype and reduced ICI benefit. This inconsistency likely reflects differences in treatment context, co-mutation status, mutation versus protein-loss definitions, and prior VEGF exposure, and highlights the need for context-dependent interpretation rather than a binary biomarker framework.

#### 2.1.2 Epigenetic changes

Epigenetic modifications play an important role in immune resistance in RCC. Aberrant DNA hypomethylation of PHLDA2 gene can lead to its upregulation. High PHLDA2 expression correlates with adverse clinical pathological parameters and poorer prognosis in ccRCC (19). Moreover, ccRCC with high PHLDA2 expression exhibits an immunosuppressive microenvironment characterized by high infiltration of Tregs and cancer-associated fibroblasts (CAFs), and this expression pattern is associated with reduced efficacy of ICI plus anti-angiogenic combination therapy (19). These findings suggest that epigenetic changes may contribute to immune resistance by reshaping the microenvironment. However, the evidence remains insufficient. Whether PHLDA2 is a causal driver of ICI resistance or a clinically validated predictor of differential ICI benefit is still unresolved and will require confirmation in cohorts with defined histology, treatment exposure, and endpoints, as well as functional perturbation or rescue experiments, and external validation.

#### 2.1.3 Metabolic reprogramming

RCC is characterized by metabolic disorders. More than 80% of ccRCC cases show VHL gene inactivation, leading to abnormal activation of hypoxia inducible factor (HIF). Sustained HIF activation drives comprehensive metabolic remodeling, including abnormal lipid synthesis, aerobic glycolysis (the Warburg effect), and glutamine addiction (20–22). HIF upregulates key enzymes, including fatty acid synthase and stearoyl CoA desaturase, enhancing lipid synthesis (23). Multiple lipid uptake proteins, such as CD36 and SCARB1, are significantly upregulated in ccRCC, leading to marked lipid accumulation (24). Tumor cells not only gain growth advantages by reshaping lipid metabolism, but also reprogram surrounding immune cells, establishing a strong barrier against immunotherapy (25). A recent 2026 study, using single-cell and spatial transcriptomics, proposed a multicellular program (MCP) associated with immunotherapy resistance (26). This program was reported to be co-driven by TAM_APOE, ccRCC_CXCL14 tumor cells, and endothelial cells, in which ccRCC_CXCL14 cells were proposed to recruit TAM_APOE, which in turn may promote tumor lipid metabolism reprogramming and angiogenesis, forming a putative pro-tumor feedforward loop. This MCP was significantly enriched in non-responders (26). However, this mechanism is derived from a single study, has not yet been independently replicated in external cohorts and should not be regarded as an established resistance driver. Competitive cholesterol uptake by tumor cells decreases microenvironment cholesterol, inducing macrophages to polarize toward the immunosuppressive M2 type and helping tumor cells achieve immune escape (27). Furthermore, lipid metabolism is closely related to iron-dependent cell death, and ccRCC cells evade this death process through lipid remodeling (24).

Lipid metabolism reprogramming in RCC leads to immunotherapy resistance through a multi-level process. This vicious cycle is triggered by genetic mutations within tumor cells. Metabolic products and microenvironmental changes induce T cell exhaustion and tumor associated macrophages (TAMs) and myeloid-derived suppressor cells (MDSCs) accumulation (20), creating an immunosuppressive microenvironment that helps tumor cells evade immune surveillance, allowing tumors progression and further metabolic abnormalities. Ultimately a robust resistance barrier is constructed.

HIF upregulates glycolysis related genes, such as glucose transporter 1 and key enzymes (HK2, LDHA), allowing RCC cells to preferentially use glycolysis pathway to produce energy and biosynthetic precursors even under aerobic conditions. The final product, lactic acid, is pumped out through lactate transporters such as MCT4, encoded by SLC16A3 (28). Extracellular lactic disrupts antitumor immunity through multiple pathways. It reduces TME pH and inhibits CD8^+^ T-cells proliferation and killing ability; activates GPR81, converting antitumor M1 macrophages into pro-tumor M2 macrophages that secrete immunosuppressive factors (28). Lactic acid creates an environment conducive to Tregs and MDSCs aggregation and activation. Recent research reveals a more complex mechanism: lactate secreted by ccRCC cells through MCT4 not only affects immune cells, but also activates ERK signaling through GPR81, stabilizes c-MYC, and further upregulates glycolysis related genes such as LDHA. This forms a lactate driven autocrine positive feedback loop, continuously enhancing the glycolytic phenotype and immunosuppressive state (28).

Another core metabolic mechanism driving immunotherapy resistance in RCC, especially ccRCC, is amino acid metabolism reprogramming. Abnormal HIF activation caused by VHL inactivation triggers remodeling of metabolic network covering glutamine, tryptophan, arginine, and cysteine (29). Tumor cells and stromal cells in the TME compete for nutrients such as glucose and glutamine, contributing to CD8^+^ T-cell exhaustion and impaired cytotoxic function. Excessive glutamine consumption triggers TAM secretion of IL-23, which promotes Tregs proliferation and inhibits anti-tumor immune responses (20). Tumor cells overexpress indoleamine 2,3-dioxygenase 1 (IDO1), which catabolizes tryptophan into kynurenine and downstream metabolites. Kynurenine promotes Treg activation and suppresses CD8^+^ T-cell proliferation and effector function (29). Separately, uric acid, a purine catabolite released during cellular stress and tumor turnover, can impair CD8^+^ T-cell function (30). These two pathways, tryptophan-kynurenine metabolism and purine catabolism, should be considered distinct contributors to immune suppression rather than a single metabolic axis. Amino acid metabolism changes in in RCC also affect other key immune cells, transforming them from attackers to accomplices. For example, metabolic reprogramming induces TAMs to polarize towards the immunosuppressive M2 type, further suppressing T-cell activity (20). The tumor metabolic environment favors MDSCs aggregation and activation. Therefore, amino acid metabolism reprogramming in RCC is a complex network in which tumor cells cause starvation and paralysis of effector T cells through metabolic remodeling of multiple amino acids and reverse surrounding immune cells, establishing a multi-level immunotherapy resistance barrier.

#### 2.1.4 Activation of SPP1-CD44 signal axis

Compared with sensitive group, the interaction between tumor cells and immune cells mediated by SPP1-CD44 signaling are significantly increased in patients with primary drug-resistant (31). Tumor cells expressing SPP1 interact with exhausted CD8^+^ T cells expressing CD44, activating MAPK signaling, exacerbating T cell exhaustion, and driving immunotherapy resistance (31).

#### 2.1.5 Activation of SAA-CEACAM1-TIM-3 axis

A single-cell sequencing study reported that SAA^+^ tumor cells were significantly enriched in patients who did not respond to ICI plus TKI combination therapy (32). These tumor cells were proposed to interact with a VEGFA^+^CEACAM1^+^ immunosuppressive neutrophil subset through SAA signaling, forming a candidate signaling axis that may promote drug resistance (32). In animal models, pharmacological inhibition of SAA or blockade of the CEACAM1–TIM-3 axis restored treatment sensitivity and enhanced ICI plus TKI efficacy (32). On the basis of these findings, the SAA–CEACAM1–TIM-3 axis has been proposed as a candidate driver of drug resistance and a potential target for improving therapy efficacy. However, this evidence derives from a single study only; independent replication in defined clinical cohorts, with defined treatment exposure and endpoints, is required before this axis can be regarded as an established resistance mechanism or a validated therapeutic target.

#### 2.1.6 KPNA2 mediated immunosuppressive environment

High KPNA2 expression is associated with poor treatment response and is a candidate resistance marker (33). KPNA2-high tumors may recruit more immune cells, but CD8^+^ T-cells show impaired function, with decreased granzyme B and increased Treg infiltration (33). This may promote an immunosuppressive microenvironment and contribute to resistance (33). However, the cited study reports a candidate biomarker and associated immune features, not a definitive resistance indicator. Whether KPNA2 high expression can predict differential benefit from ICI treatment still requires validation in studies with defined treatment exposure, endpoints, and external validation.

#### 2.1.7 TK1 and immune cell dysfunction

Thymidine kinase 1 (TK1) is a key enzyme in DNA precursor synthesis and has been reported to be associated with tumor immune evasion (34). In associational analyses, TK1 is associated with infiltration of immunosuppressive cells, including PD-L1^+^ macrophages and Tregs, and with immunosuppressive molecules, including TGFB1, CXCL8, IL10 and MMP1. These cells and molecules are important regulatory factors of antitumor immunity, and their infiltration or overexpression may induce immune suppression (34). However, current evidence does not establish a direct functional mechanism. Functional perturbation or rescue experiments and clinical validation are needed before TK1 can be considered a predictive biomarker of ICI benefit.

Even when focusing on tumor cell-intrinsic mechanisms, tumor cells do not operate in isolation. Metabolic reprogramming and epigenetic changes are adaptive changes within tumor cells, but these internal changes ultimately lead to immunotherapy resistance through systematic reshaping of the external TME. Moreover, molecules such as KPNA2 (33) and SAA (32) not only alter cancer cells, but also reshape the entire microenvironment, recruiting or transforming immune cells to establish a vicious cycle favorable for tumor survival.

### 2.2 The TME related mechanisms

Resistance of RCC to immunotherapy, particularly ICIs, is extremely complex, with the core lying in profound remodeling of the TME and establishment of immune suppression. This process is not caused by a single factor but by a complex network formed by synergistic interactions among multiple cells types, signaling molecules, and metabolic pathways.

#### 2.2.1 Recruitment of immunosuppressive cells

Tumors can reprogram or recruit various immunosuppressive cells, including Tregs and myeloid cells such as MDSCs and TAMs, constructing an immunosuppressive microenvironment.

Myeloid cells are the most abundant immune cells population in the TME. In RCC, they are often hijacked by tumors, transforming from immune attackers to inhibitors. Continuous type-I and type-II interferon (IFN) signaling in myeloid cells, especially macrophages, drives multiple ICI resistance mechanisms (35). In RCC tumors, the CD8^+^LAG3^+^ T-cell subset is the main source of IFN-γ in the TME, and these cells secrete IFN-γ that acts on surrounding macrophages (35). Upon receiving IFN-γ signals, macrophages strongly upregulate genes encoding inhibitory ligands and immune checkpoints (35). Notably, this upregulation is far more pronounced in macrophages than in renal cancer cells themselves (35), explaining why resistance is tumor-extrinsic (35). These macrophages become potent immunosuppressive cells, directly inhibiting T-cell killing activity. This mechanism has been validated in clinical trial cohorts. IFN-γ signaling in myeloid cells, rather than in tumor cells, effectively predicted primary resistance to first-line ICI combined with anti VEGF therapy (35). The core pathway can be summarized as follows: intratumoral CD8^+^ T cells secrete IFN-γ, which acts on intratumoral macrophages, driving them to highly express inhibitory molecules that suppress T-cell function, ultimately leading to ICI treatment failure. This discovery elucidates a new resistance mechanism and suggests that IFN-γ signaling in myeloid cells, especially macrophages, represents a potential resistance biomarker and therapeutic target.

TAMs are major drivers of immune suppression. The TAM subgroup with high secreted phosphoprotein 1 (SPP1) expression is enriched in ICI-resistant RCC (36). Patients with high SPP1^+^ TAM infiltration have poor response to immunotherapy and extremely poor prognosis (36). SPP1^+^ TAMs exhibit an immunosuppression and protumor phenotype, and mediate resistance by inducing CD8^+^ T cell dysfunction and terminal differentiation (36). Therefore, SPP1^+-^TAMs drives immunotherapy resistance through CD8^+^ T-cell dysfunction (36).

MDSCs are associated with disease progress in RCC. Compared with normal populations, MDSC numbers are significantly increased, indicating impaired immune function (37). PMN-MDSC and M-MDSC levels increase with tumor diameter and clinical stage progression (37). MDSCs inhibit antitumor immunity through arginase 1 (Arg1), inducible nitric oxide synthase (iNOS), reactive oxygen species (ROS)-mediated pathways, and other recently discovered pathways (38). However, the cited study compared immune-cell measures in RCC patients and controls and related them to disease characteristics. It did not establish MDSCs as a prognostic biomarker for ICI treatment. Whether MDSCs can predict differential ICI benefit remains to be validated in cohorts with defined treatment exposure and endpoints.

In addition, neutrophil extracellular trap (NET) formation is associated with RCC progression and ICI resistance, with serum amyloid A1 (SAA1) as a critical mediator. Mechanistically, SAA1 binding to TLR2 activates NF-κB signaling, which promotes NET formation (39). These NETs act as physical barriers preventing contact between CD8^+^ T-cells and tumor cells, and promote CD8^+^ T-cell exhaustion, facilitating tumor immune evasion and resistance to immunotherapy (39).

Tregs are lymphocytes that regulate and inhibit overreaction of other immune cells. Infiltration of a large number of Tregs into tumor tissue is usually associated with poor prognosis. Their abundance leads to immunosuppression reprogramming of the tumor environment, favoring tumor growth and low drug efficacy. Tregs suppress antitumor immunity through inhibitory cytokines such as IL-10, TGF-β, and IL-35, and through functional molecules such as CTLA-4 and PD-1 (40). In addition, Tregs can release granzyme B, a serine protease that induces target-cell apoptosis, inhibiting antitumor activity of effector T cells and NK cells, and promoting tumor progression and immunotherapy resistance (40). TGFBI is an extracellular matrix protein induced by TGF-β and associated with Treg regulation. TGFBI promotes Treg infiltration and enhances CCL22 expression, facilitating formation of an immunosuppressive microenvironment in ccRCC, thereby promoting tumor progression and treatment resistance (41). Targeting TGFBI or Tregs may therefore provide a new strategy for improving the immune microenvironment and prognosis of RCC patients.

#### 2.2.2 Hypoxia and inflammatory signaling pathways

Hypoxia and inflammatory signaling pathways synergistically reshape tumor microcirculation, constructing a physical and chemical immune shelter. They are drivers of resistance to renal cancer immunotherapy. Chronic inflammation induced signaling pathways can drive immunotherapy resistance in RCC. RCC evolves within a renal microenvironment characterized by chronic inflammation (42). Sustained activation of NF-κB/STAT3 and HIF/VEGF signaling, as well as chronic of IFN-γ signaling, not only promotes angiogenesis, but also fundamentally reshape the immune microenvironment toward an immunosuppressive state (42).

Hypoxia-induced signaling also plays an important role in immunotherapy resistance. As noted above, RCC, despite with abundant vasculature, suffers from chronic hypoxia due to VHL/HIF pathway activation which produces structurally aberrant, poorly perfused vessels. Under low-oxygen conditions, effector T cells become less effective and may undergo apoptosis or exhaustion. Hypoxia can also upregulate the expression of PD-L1 on tumor cells, triggering immunosuppressive signals and inhibiting T-cell activation (43). Meanwhile, hypoxia facilitates adenosine accumulation in the TME, strongly inhibiting T-cells and NK-cells activity through A2A receptor (43). Moreover, RCC exhibits Warburg-like metabolism, producing lactate and leading to acidic extracellular pH. Low pH inhibits T-cell receptor signaling and cytotoxic function, and impairs dendritic cell maturation, leading to an immune-cold phenotype (44). Hypoxia causes macrophages to polarize toward the immunosuppressive M2 phenotype, while an acidic environment inactivates effector lymphocytes and antigen-presenting cells, jointly establishing a strong barrier for immunotherapy. Overall, these hypoxia-driven events establish a multifaceted immune suppression network, greatly limiting ICI efficacy in RCC.

#### 2.2.3 Upregulation of alternative immune checkpoints

Upregulation of alternative immune checkpoints, such as lymphocyte activation gene-3 (LAG-3), T cell immune receptor with Ig and ITIM domains (TIGIT), T-cell immunoglobulin and mucin domain containing protein-3 (TIM-3), is considered a candidate mechanism of acquired drug resistance in RCC after PD-1/PD-L1 blockade (12, 45). This upregulation may serve as a backup pathway for tumor immune evasion and can attenuate, though not necessarily abolish, the benefit of immunotherapy. These checkpoints are often co-expressed with PD-1 on tumor infiltrating T cells, forming a complex and multi-level immunosuppressive network (45). When treated with PD-1 inhibitors, some T cells are reactivated, but others upregulate LAG-3 or TIM-3. In RCC tumor-infiltrating lymphocytes (TILs), PD-1 and LAG-3 are the most common co-expression combination, and PD-1 blockade significantly upregulates LAG-3 expression, whereas TIM-3 regulation appears more context-dependent (46). This suggests a dynamic adaptive process driven by the TME, in which tumor cells and infiltrating immune cells may compensatorily upregulate other inhibitory molecules after a single immune checkpoint is suppressed, re-establishing the immune suppression barrier (22, 45). However, the cited PD-1/LAG-3 study includes ex vivo/*in vitro* observations in surgical samples. It supports a candidate adaptive mechanism rather than demonstrating that this mechanism entirely negates clinical ICI benefit. Functional perturbation, rescue experiments, and external validation are still needed to clarify its clinical predictive value.

#### 2.2.4 Physical barriers and matrix barriers

Beyond cells and molecules, the physical structure of the TME can impede immune cell attack.

Renal cancer, especially ccRCC, is characterized by abnormal proliferation of blood vessels with functional disorders. This chaotic vascular network fails to effectively deliver oxygen, and expression of adhesion molecules such as ICAM-1/VCAM-1 on endothelial cells is downregulated (42). This prevents T cells in the blood from “grasping” the vessel wall and penetrating into tumor tissue. As a result, T cells are excluded from the tumor core and cannot enter cancer cells.

In ccRCC TME, cancer-associated fibroblasts (CAFs) drive a self-sustaining cycle of immune evasion and therapeutic resistance through interactions between cells and molecules (47). CAFs promote tumor growth through metabolic reprogramming and regulate immune suppression by driving M2 polarization of TAMs and amplification of Tregs, promoting a multilayered immune suppression network (47). CAFs are the most active stromal cells in the TME. The extracellular matrix (ECM) is mainly secreted by CAFs and composed of collagens, laminins, glycoproteins, fibronectin, proteoglycans, and polysaccharides (48). Under the action of CAFs, the ECM undergoes severe remodeling, becoming abnormally stiff and dense, limiting T-cell movement (42). High expression of the ECM component COL6A2 is associated with increased infiltration of immunosuppressive cells and resistance to immunotherapy (49). This matrix barrier directly hinders T-cells migration to the deep part of the tumor. In advanced RCC, the TME typically manifests as one of three immune phenotypes, including immune-infiltrated, immune-excluded, or immune-desert (42). Each is defined by different immune cell characteristics, spatial patterns, and functional states. The predictive value of traditional biomarkers such as PD-L1 expression, which are effective in melanoma and lung cancer, is very limited in RCC (50). This further underscores that resistance mechanisms in RCC result from systemic microenvironment action, rather than determination by a single molecule.

Therefore, multiple TME factors, including recruitment of immunosuppressive cells, hypoxia and inflammatory signaling pathways, upregulation of alternative immune checkpoints, and physical barriers, synergistically drive RCC resistance to immunotherapy.

### 2.3 Host related mechanisms

#### 2.3.1 Gut microbiome dysbiosis

Antibiotics may reduce ICI efficacy by inducing gut microbiota dysbiosis (51, 52). Antibiotic use can lead to ecological imbalance of bacteria. Microbiota imbalance may disrupt the MAdCAM-1–α4β7 axis, promote recruitment of T regulatory 17 (Tr17) cells producing immunosuppressive IL-17 into tumors, and contribute to ICI resistance (53). Retrospective studies consistently indicate that RCC patients who use antibiotics before or during immunotherapy have lower ORRs and shorter PFS and OS (54, 55). However, these retrospective associations are subject to confounding by indication, such as infection severity, disease burden, performance status, and concomitant medications, and should not be interpreted as causal. Prospective studies or causal inference approaches are needed to confirm whether antibiotics causally reduce ICI efficacy.

Specific bacterial species, such as *Clostridium hathewayi*, are significantly enriched in RCC patients who did not respond to ICI treatment and are associated with antibiotic use and resistance (51). Fecal microbiota transplantation (FMT) has shown preliminary promise in augment immunotherapy in phase I/II clinical trials (52, 56).

#### 2.3.2 Obesity paradox

In RCC, the impact of obesity on ICI efficacy presents a paradox, with complex mechanisms that promote drug resistance and contradictory evidence that it may enhance efficacy (57). Recent research reveals core mechanisms of obesity-driven ICI resistance, including inflammation and IL-1β- driven resistance, metabolic reprogramming and immune suppression, and multicellular interaction programs.

Under obesity, persistent low-grade chronic inflammation occurs (57). Levels of the pro-inflammatory cytokine interleukin-1β (IL-1β) are significantly elevated in obese RCC patients and mouse models (58). High IL-1β promotes aggregation of immunosuppressive cells such as MDSCs within tumors, inhibiting CD8^+^ T-cells antitumor activity and reducing response to PD-1 inhibitor therapy (58). The response rate of obese mice to PD-1 treatment is significantly lower than that of non-obese mice (44% vs 73%) (58). Neutralizing IL-1β in obese mice increased the therapeutic response rate to 58% and reduced intratumoral MDSC accumulation (58).

RCC, especially ccRCC, is essentially a metabolic disease. Obesity profoundly alters the TME by releasing large amounts of fatty acids, adipokines, such as leptin and resistin, and inflammatory factors through adipose tissue (59). Obesity increases tumor promoting factors such as leptin, resistin, IL-1β, IL-6 and VEGF, while protective adiponectin and Omentin-1 decrease (59). This imbalanced microenvironment promotes angiogenesis, matrix remodeling, and immune escape. Obesity comprehensively reshapes the immune microenvironment toward an immunosuppressive state by recruiting MDSCs, polarizing TAMs, inducing T cell depletion, and increasing Tregs (59).

A recent 2026 study proposed a multicellular program (MCP) associated with ICI resistance in ccRCC using single-cell and spatial transcriptome techniques (26). The program was characterized by enhanced lysosomal activity, activation of adipogenic signals, and rewired fatty acid metabolism (26). The study proposed a putative vicious cycle in which tumor cells recruit specific types of TAM_APOE macrophages by secreting the chemokine CXCL14, which in turn may promote tumor lipid metabolism reprogramming and angiogenesis, forming a positive feedback loop that promotes tumor growth and contribute to treatment resistance and poor prognosis (26). This provides a detailed intercellular interaction framework that may help explain how metabolic reprogramming within the TME contributes to ICI resistance, a mechanism potentially relevant to obesity-associated resistance. As this model is based on a single study without independent replication or functional perturbation, it should currently be considered hypothesis-generating rather than established.

Although these mechanisms suggest that obesity may promote ICI resistance, clinical observations have found contradictory results. In metastatic RCC, high BMI (obesity) is often associated with longer OS after ICI treatment (57). For example, the ICI response rate in obese patients can reach 50.0%, while in non-obese patients it is only 30.28% (57). However, this benefit is not consistent across all studies. After adjusting for known prognostic risk scores such as IMDC, the survival advantage associated with BMI weakens or disappears (60). This suggests that BMI may be a confounding factor, or its role may be overshadowed by other prognostic factors. In addition, obese RCC patients receiving anti-PD-1 based treatment have been reported to have shorter PFS and OS (58). This contradiction may stem from patient heterogeneity, limitations of BMI as an obesity indicator, inability to distinguish muscle and fat mass, and different treatment options (57). In summary, obesity constructs a complex immunosuppressive microenvironment in RCC through IL-1β-driven inflammation, metabolic reprogramming, and multicellular interactions, forming an important foundation for ICI resistance. However, the “obesity paradox” suggests that this resistance does not always translate into adverse clinical outcomes, and the prognostic value of BMI is highly context-dependent. Current research focused on identifying biomarkers that can distinguish obesity-related resistant individuals from beneficiaries, such as visceral fat and inflammation levels, to achieve more accurate patient stratification.

To clarify the evidence, it is useful to distinguish demonstrated explanations from hypotheses. The IL-1β-driven inflammatory mechanism is supported by clinical and murine data. Obese RCC patients and mouse models show elevated IL-1β, which promotes MDSC accumulation and reduces PD-1 inhibitor response, and neutralizing IL-1β restores therapeutic response and reduces intratumoral MDSCs in obese mice (58). In contrast, explanations involving adipose-derived leptin/resistin signaling, adiponectin depletion, and microbiome-mediated obesity–immune interactions remain largely hypothetical, with limited prospective validation and insufficient stratification by treatment line. The prognostic value of BMI itself is highly context-dependent: some studies report improved OS with high BMI after ICI treatment, whereas others report shorter PFS and OS in obese patients receiving anti-PD-1-based therapy (57, 58). In RCC, after adjustment for IMDC risk scores, the apparent survival advantage of high BMI weakens or disappears (57). Therefore, obesity-related factors should currently be regarded primarily as stratification variables, while specific mechanistic targets such as IL-1β require prospective clinical validation before being considered direct therapeutic targets. Future studies should distinguish visceral adiposity and inflammation levels, and should also account for treatment line, cancer type, and ICI regimen, to identify obesity-related resistant versus beneficiary subpopulations.

#### 2.3.3 Age

Age affects tumor biological characteristics and immune microenvironment, potentially leading to differences in ICI response and resistance across age groups. However, the direct causal relationship between age and resistance is still being explored. Age can affect the gene mutation spectrum of RCC tumors, and these genetic changes may affect ICI efficacy and resistance by affecting tumor immunogenicity. A large-scale genomic analysis of 3,360 RCC patients found that MUC4 and TP53 mutations were more common in young patients (<45 years), while elder patients (>75 years) were enriched with VHL, PBRM1, and TTN gene mutations (61). Although these age-related gene changes were associated with OS and tumor immune cell infiltration, no significant predictive relationship was found with ICI treatment response in this study, possibly due to limited sample size for ICI response analysis (61). This means that these genetic changes may not directly lead to ICI resistance, but their shaping effect on the TME may indirectly affect treatment efficacy.

Immune aging is also a core factor affecting ICI efficacy. An analysis of two clinical trials, CheckMate 214 and JAVELIN Renal 101, involving 964 patients receiving ICIs, revealed a decreasing trend in efficacy with age (62). For patients aged ≤ 60 years, immunotherapy was associated with better overall survival (OS; HR = 0.70); for those over 60 years, OS benefit was marginal (HR = 0.82) (62). Young patients exhibit increased immune cell infiltration, enhanced tumor immunogenicity, and improved immune responses (62). With aging, immune function declines and T-cell pools diversity decreases, weakening the ability of ICIs to activate antitumor immune responses. A study of cancer patients aged≥80 years provides additional evidence (63). Elderly RCC patients have a lower proportion of PD-L1 positive tumors (9.0% vs 19.4%) compared with younger patients (63). However, this observation does not necessarily imply that PD-L1 targeting agents have fewer actionable targets for immune activation in elderly patients, nor does it directly indicate greater resistance. This is particularly important given the limited predictive utility of PD-L1 in RCC. With increasing age, an unfavorable environment for immunotherapy may develop, characterized by immunosenescence and distinct mutational landscapes, which may increase the risk of resistance or attenuate efficacy. The impact of age on immunotherapy resistance is unlikely to be driven by a single age-related gene. This remains a hypothesis requiring dedicated validation.

#### 2.3.4 Sex

Sex differences shape different tumor immune microenvironment, directly affecting immunotherapy effect and resistance mechanisms. In RCC, the impact of sex on ICI resistance is not simply about “which sex has better outcomes”, but reflects fundamental differences in the tumor immune microenvironment. A large-scale retrospective study published in 2025 analyzed 563 advanced RCC patients receiving different regimens (64). Among patients receiving dual immunotherapy (IO-IO) with nivolumab and ipilimumab, the median PFS in females was significantly shorter than in males (3.85 months vs. 10.2 months) (64). Among patients receiving nivolumab monotherapy, shorter PFS was also observed in females (3.76 months vs. 7.30 months) (64). Further analysis showed that sex was an independent factor for shorter PFS in the IO-IO (p=0.0340) and nivolumab monotherapy groups (p=0.0322) (64). In contrast, no significant PFS difference was observed between sexes in the IO-TKI or TKI monotherapy group (p>0.05) (64). This indicates that the impact of sex on therapeutic efficacy is mainly related to immunotherapy itself, rather than targeted drugs. The difference in clinical outcomes is attributed to varying immune cells states in the TME, including androgen-driven depletion of CD8^+^ T-cells in male RCC patients and insufficient immune cell infiltration in female RCC patients.

Single-cell sequencing has shown that CD8^+^ T-cell infiltration in the TME of male RCC is higher than in females (65). However, most of these T cells are exhausted and dysfunctional, unable to effectively kill tumor cells (65). Androgens can induce dysfunction and exhaustion of CD8^+^ T-cells through the androgen receptor axis, forming an immunosuppressive microenvironment (65). *In vitro* and *in vivo* experiments have shown that the dysfunction and exhaustion of CD8^+^ T-cells in male TME are induced by androgen (65). Male RCC patients with higher serum androgen levels have a poorer prognosis after immunotherapy (65). In animal experiments, androgen receptor inhibitors activate tumor-infiltrating CD8^+^ T cells and enhance immunotherapy efficacy, indicating that combining androgen receptor inhibitors with immunotherapy may be promising for male RCC patients (65).

However, tumor-infiltrating immune cells such as CD8^+^ T cells are significantly lower in female patients with advanced RCC than in males (64). This may be the direct cause of poor immunotherapy response. Male RCC patients mainly face androgen-driven T-cell exhaustion, while female RCC patients are trapped in insufficient immune cell infiltration. Understanding these two distinct immunobiological patterns may help guide sex-specific immunotherapeutic strategies.

## 3 Novel therapeutic strategies and clinical progress in overcoming resistance

### 3.1 ICI combined with anti-angiogenic TKI

We distinguish three clinical settings when discussing resistance: initial treatment to prevent or delay resistance; subsequent treatment after documented ICI progression; and experimentally demonstrated re-sensitization, in which previously resistant tumors regain sensitivity to the same class of ICI. Primary resistance denotes progression without initial benefit, wheras acquired resistance denotes progression after an initial response. Evidence from one setting does not automatically apply to another.

In RCC, ICI plus TKI is the core strategy for advanced RCC and represents a strategy to prevent or delay resistance relative to sunitinib monotherapy (5–7). The combination is not simply a superposition, but is based on a biological rationale to overcome resistance at multiple levels. The highly immunosuppressive TME is the main reason for ICI resistance (66). TKIs, such as axitinib, cabozantinib, and lenvatinib transiently normalize tumor vasculature by inhibiting angiogenesis, improving immune cell infiltration and function and increasing sensitivity to immunotherapy (67, 68). TKIs can also reduce immunosuppressive myeloid recruitment, creating favorable conditions for ICI activity (68). PD-1/PD-L1 inhibitors reactivate suppressed T cells, enabling them to recognize and attack tumor cells (69). This rationale has been clinically validated in the first-line setting by KEYNOTE-426 (Pembrolizumab plus Axitinib) (5), CheckMate 9ER (Nivolumab plus Cabozantinib) (6), and CLEAR study (Lenvatinib plus Pembrolizumab) (7), each demonstrating PFS and OS superiority over sunitinib. These trials enrolled ICI-naive patients and therefore establish the activity of combination regimens as initial therapy. They do not demonstrate restored sensitivity in patients with documented progression on prior ICI therapy, nor do they alone establish pharmacological synergy.

Despite significant therapeutic effects, resistance remains a serious challenge. Understanding resistance mechanisms and developing relay or combination strategies is the core of future research. High expression of thymidine kinase 1 (TK1) is significantly associated with poorer PFS in patients receiving ICI+TKI therapy. The mechanism may involve T-cell dysfunction caused by high TK1 expression, manifested as an increased in PD1+CD4^+^ T-cells, PD-L1+macrophages, and Tregs (34). Tumor cells can also evade attacks by activating alternative signaling pathways. For example, axitinib resistance may be associated with increased nuclear protein 1 (NUPR1) or decreased insulin receptor (INSR) levels (70). Cabozantinib resistance may be caused by secretion of non-VEGF angiogenic factors such as PDGF-BB and IL-8 (70). Lenvatinib resistance may involve activation of alternative pathways such as EGFR-PAK2-ERK (70).

Whether adding a TKI can reverse established ICI resistance is a distinct question requiring randomized evidence in patients who have progressed on prior ICI therapy. Two phase 3 trials have addressed this. In CONTACT-03, atezolizumab plus cabozantinib did not improve PFS over cabozantinib alone in patients who had progressed on prior ICI therapy, and the combination increased toxicity (71). In TiNivo-2, tivozanib plus nivolumab did not improve PFS over tivozanib monotherapy in patients who had progressed after one or two prior lines including an ICI (median PFS 5.7 vs 7.4 months; HR 1.10) (72). These negative results indicate that, in advanced RCC, ICI rechallenge after prior ICI progression should be discouraged, and that the benefit of ICI+TKI combinations is largely confined to the first-line setting. Importantly, these clinical results do not contradict the biological rationale that VEGF inhibition can modulate antitumor immunity. They indicate that preclinical synergy does not necessarily translate into clinical re-sensitization after ICI resistance.

In the first-line setting, ICI plus TKI may delay progression or improve outcomes relative to sunitinib monotherapy, but this does not demonstrate reversal of established ICI resistance. Combination therapy is not a definitive solution and may introduce new resistance challenges. For patients who progress after ICI-based therapy, the relevant question is not ICI rechallenge but later-line antitumor activity through mechanisms independent of ICI sensitivity. In the randomized phase 3 LITESPARK-005 trial, patients who had previously received immune checkpoint inhibitor and VEGF/VEGFR-directed therapy were assigned to belzutifan, a HIF-2α inhibitor, or everolimus. Belzutifan improved PFS and objective response versus everolimus, although OS did not differ significantly at the reported interim analysis (73). This represents later-line antitumor activity rather than restored ICI sensitivity. Final OS maturity, longer follow-up, and validation in diverse populations are required before belzutifan can be positioned as a definitive standard. Current and future research is shifting toward identifying new resistance mechanisms, validating reliable predictive biomarkers, and developing next-generation combination strategies.

### 3.2 Dual ICI therapy

A core reason for resistance to single immunotherapy such as PD-1 inhibitors is the presence of multiple inhibitory pathways in the TME. CTLA-4 inhibitors and PD-1 inhibitors have different mechanisms of action. The former mainly affects initiation and activation of early T cells in lymph nodes, while the latter mainly restores exhausted T-cell activity in the TME. Their combination can more effectively activate antitumor immune responses.

However, dual ICI therapy still faces the challenge of resistance. Approximately 20-25% of advanced RCC patients exhibit primary resistance to first-line nivolumab plus ipilimumab (74, 75). A worldwide multicenter retrospective real-world study involving 1709 metastatic clear cell renal cell carcinoma patients from 22 countries and 72 centers, the patients received first-line immune-oncology combination (76). The research results show that the overall population has a primary resistance rate of 19% (76), and Nivolumab plus ipilimumab showed the highest primary resistance rate of 27% (76). Preclinical vaccine combining anti-PD-1 and anti-CTLA-4 antibodies have shown antitumor activity in mice renal cell carcinoma model (77), but these findings should not be extrapolated directly to human clinical benefit. Outcomes after subsequent treatment for patients with primary immunotherapy resistance are usually unsatisfactory. Compared with those without primary resistance, patients who develop primary resistance have significantly shorter survival after second-line treatment (78). These findings highlight that even with dual checkpoint blockade, a substantial proportion of patients derive limited benefit, and negative or null results from individual trials should be interpreted alongside positive ones when evaluating combination strategies.

Current efforts to address resistance in dual immunotherapy focus on prediction and overcoming. A retrospective multicenter study of 89 patients receiving first-line nivolumab plus ipilimumab, including 23 primary-resistance events, identified sarcomatoid differentiation and pretreatment serum lactate dehydrogenase (LDH) ≥ 174 U/L as independent factors associated with primary resistance to dual ICI therapy (75). In that study, primary resistance was defined as disease progression within 3 months of treatment initiation, and the LDH cutoff of 174 U/L was derived by ROC analysis in the same cohort and has not been externally validated. Because this was a retrospective, within-regimen analysis, it does not establish which alternative regimen would improve outcomes for patients with these characteristics and does not justify avoiding dual ICI solely on the basis of these features. This interpretation must be reconciled with the exploratory 5-year sarcomatoid subgroup analysis of CheckMate 214, in which nivolumab plus ipilimumab demonstrated substantial benefit versus sunitinib, with an ORR of 60.8% vs 23.1%, a median OS of 48.6 vs 14.2 months (HR 0.46), and a median PFS of 26.5 vs 5.5 months (HR 0.50) (3). A within-regimen association with early progression and a relative benefit against a comparator are different questions and can coexist. However, this subgroup analysis was *post hoc* and exploratory and was not a dedicated randomized comparison of ICI-TKI versus dual ICI. Therefore, sarcomatoid differentiation and LDH ≥174 U/L are now presented as candidate predictors of primary resistance to dual ICI require contextual interpretation and prospective validation, rather than as standalone criteria for treatment selection.

It should be emphasized that the negative results of CONTACT-03 and TiNivo-2 (71, 72)discourage sequential use of ICI rechallenge after prior ICI progression. They do not exclude the possibility that dual ICI therapy, or other ICI-based combinations, may benefit selected patients in the first-line setting or in distinct resistance contexts. The critical task is therefore to distinguish primary resistance from acquired resistance, and to define which patients may still derive benefit from ICI-containing regimens versus those who require ICI-independent mechanisms such as HIF-2α inhibition (73).

Dual immunotherapy is an established first-line strategy that may delay or partially overcome resistance to single immunotherapy, but primary resistance occurs in approximately 20-25%. Current focus has shifted toward identifying candidate predictive biomarkers, such as sarcomatoid differentiation and high LDH, that require contextual interpretation and prospective validation, and toward further enhancing efficacy through combination microenvironment remodeling therapy and next-generation immunomodulatory agents.

### 3.3 Novel immunotherapeutic strategies regulating the TME

The RCC TME is a highly vascularized, immune infiltrated, but functionally inhibited complex ecosystem (1). Targeting the TME to delay, partially overcome, or bypass immunotherapy resistance is an active research field. Various new combination strategies are being explored, but these strategies are in preclinical or early clinical stages. The most promising strategic directions include the following:

#### 3.3.1 Targeting tregs

Tregs are key immunosuppressive cells in the TME, suppressing immunity through direct cell contact and inhibition of cytokine secretion (79). Emerging strategies to reprogram the TME include CD25-blocking antibodies, metabolic modulators targeting Treg stability, and cell therapies or CRISPR-based Tregs regulation, all showing promise (79).

#### 3.3.2 Targeting MDSCs

MDSCs are another type of immunosuppressive cells. Sustained elevation of monocytic myeloid-derived suppressor cells (M-MDSCs) levels in peripheral blood is associated with ineffective anti-PD-1 therapy (80). Combined blockade of PD-1 and IL-8, a key chemokine recruiting MDSCs, tends to suppress tumor growth in the next-generation patient-derived xenograft (NG-PDX) model (80).

#### 3.3.3 Reprogramming TAMs

TAMs are abundant in the RCC microenvironment, usually manifesting as the pro-tumorigenic M2 type, and are correlated with T-cell exhaustion (81). Researchers have developed dual-cytokine-engineered macrophages that co-deliver IL-12 and CXCL-9 to reprogram TME and enhance anti-PD-1 responsiveness (81). *In vitro*, engineered macrophages polarize M2-like TAMs into antitumor M1 phenotype, secrete CXCL-9 to recruit cytotoxic T cells, and release IL-12 to amplify T/NK cell activation (81). In animal models, intravenous injection of tumor-targeting engineered macrophages reshapes the TME by increasing CD8^+^ T-cells, dendritic cells, and NK cells, while reducing immunosuppressive Tregs and MDSCs. Compared with anti-PD-1 monotherapy, the combination achieved 2.5-fold tumor growth inhibition (81).

#### 3.3.4 Epitranscriptomic regulation (targeting NSUN5 circPPAP2A axis)

NSUN5 is a key m5C methyltransferase that modifies circPPAP2A, ultimately activating HIF-2α signaling and creating an immunosuppressive microenvironment in ccRCC (82). In preclinical models, knockdown of NSUN5 or circPPAP2A synergized with anti-PD-1 therapy, inhibited tumor growth and restored antitumor immunity (82).On the basis of these preclinical findings, targeting NSUN5 or circPPAP2A has been proposed as a candidate combination strategy to delay, partially overcome, or bypass resistance to anti-PD-1 monotherapy. However, this evidence is preclinical and derived from a single study; clinical validation, including confirmation in patient cohorts with defined treatment exposure and endpoints, is required before this axis can be considered a clinically actionable target.

#### 3.3.5 Targeting SAA1

Serum amyloid A1 (SAA1) is a key mediator of anti-PD-1 resistance in RCC. Mechanistically, SAA1 induces NET formation by binding to toll-like receptor 2 (TLR2) and activating NF-κB signaling. These NETs act as physical barriers preventing direct contact between CD8^+^ T-cells and tumor cells and promote CD8^+^ T-cell exhaustion, facilitating tumor immune evasion and resistance to immunotherapy (39). Therefore, targeting SAA1 is a candidate strategy for delaying or bypassing immune resistance in RCC.

#### 3.3.6 Targeting karyopherin alpha 2

High KPNA2 expression is associated with the immunosuppressive microenvironment and resistance to TKI and ICI combination therapy (33). In tumors with high KPNA2 expression, although TILs increase, CD8^+^ T-cells show impaired function and reduced granzyme B expression (33). This makes KPNA2 a candidate biomarker for guiding treatment selection in advanced RCC (33).

#### 3.3.7 AlloCAR70-NKT cell therapy

Researchers have developed allogeneic CD70 directed CAR-engineered invariant natural killer T cells (AlloCAR70-NKT) from hematopoietic stem cells and progenitor cells (83). AlloCAR70-NKT cells exhibit strong cytotoxicity against RCC cells and selectively target the TME through T-cell receptor (TCR) recognition. Additionally, they eliminated CD70^+^ host allogeneic reactive T cells, promoting treatment persistence. AlloCAR70-NKT cell therapy has dual functions of targeting tumors and the TME, as well as eliminating allogeneic reactive T cells, supporting clinical investigation for the treatment of metastatic RCC (83).

At present, these strategies are in the preclinical or early clinical research stage, but they provide a roadmap for overcoming immunotherapy resistance. The core of future efforts is no longer simply to activate the immune system alone, but to select the most likely effective combination regimen through deep understanding of each patient’s unique TME, such as specific immunosuppressive cell populations, activated signaling axes, or aberrant metabolic states.

### 3.4 Combination of radiotherapy and immunotherapy

RCC has historically been regarded as relatively radioresistant, but this view has been challenged by modern precision radiotherapy techniques such as stereotactic body radiotherapy (SBRT), which can deliver high doses with high precision and achieve durable local control in selected lesions (84). Radiotherapy combined with immunotherapy is an investigational strategy in RCC, based on the rationale that radiotherapy can modulate the TME and potentially enhance immunotherapy efficacy (85, 86). However, evidence supporting this strategy should be interpreted with attention to distinct outcomes involved, that is local control of an irradiated lesion, abscopal activity, and systemic re-sensitization to immunotherapy. Local control refers to elderly patients who have remained stable for nearly five years after SBRT and are now considered as evidence of persistent local control in selected cases, rather than evidence of systemic sensitization. Abscopal activity has been described as an uncommon and incompletely understood phenomenon, primarily supported by preclinical data and case reports. The current evidence of systemic re sensitization has not been confirmed.

Radiotherapy is not merely a local treatment. Preclinical and early clinical studies suggest that irradiation of one tumor can induce systemic immune effects that may lead to regression of non-irradiated distant metastases, a phenomenon known as the abscopal effect (87, 88). This remains uncommon and incompletely understood, and case reports have described such regression in RCC patients who progressed after immunotherapy (87). In a mouse kidney cancer model, radiotherapy combined with targeted therapy and immunotherapy significantly enhanced therapeutic effect (88). Mechanistic analysis showed increased CD8^+^ T-cells infiltration and decreased CD163^+^ macrophages at non-irradiated sites, and depletion of CD8^+^ T-cells reduced efficacy, supporting an immune-mediated systemic component (88).

Prospective evidence should also be considered. NIVES was a single-arm phase II study of SBRT plus nivolumab in patients progressing after antiangiogenic therapy, not in patients with confirmed ICI resistance. Its ORR was 17%, failing to meet the prespecified 40% benchmark against a 25% historical control (89). Thus, while it adds to the uncertainty about general systemic synergy, it does not inform whether SBRT can overcome systemic ICI resistance, as that hypothesis was not tested in an ICI-resistant population.

What’s more, local control of oligo-progression is a distinct endpoint from systemic re-sensitization. For example, an 80-year-old patient with RCC maintained radiological and clinical stability of an irradiated lesion for nearly five years after SBRT, despite long-term discontinuation of systemic immunotherapy (84). This case supports the potential for durable local control in selected patients, but it does not by itself demonstrate reversal of systemic ICI resistance.

In summary, radiotherapy is no longer viewed solely as a local treatment. It may also exert immunomodulatory effects in selected RCC settings. However, current evidence supports a context-dependent, hypothesis-generating role rather than a general systemic resistance-reversal effect. Prospective studies with well-defined populations, appropriate comparators, and separate reporting of local control, abscopal activity, and systemic re-sensitization are needed.

### 3.5 Gut microbiota modulation

Intestinal microbiota can affect T cells differentiation and function through metabolites such as short-chain fatty acids, thereby influencing antitumor immune responses and immunotherapy efficacy (90). Therefore, regulating the gut microbiota is a candidate strategy to delay, partially overcome, or bypass immunotherapy resistance. Relevant research has progressed from mechanism exploration to early-phase clinical evaluation (90, 91). Oral administration of *Bifidobacterium longum* 420 as a cancer vaccine enhanced ICI efficacy and showed survival benefit in RCC mouse models (77). In addition, certain bacteria strains, such as *Akkermansia muciniphila* and *Clostridium butyricum*, may be associated with better immunotherapy responses (91). Supplementing specific beneficial bacterial strains has emerged as a potential precision intervention strategy. For example, *Clostridium butyricum* (CBM 588) has attracted considerable interest as a probiotic (92). In a randomized phase I trial of 30 treatment-naive patients with metastatic RCC, the addition of CBM588 to nivolumab plus ipilimumab was associated with a favorable PFS signal (12.7 vs 2.5 months, HR 0.15, p=0.001) (92). However, this PFS result was a secondary outcome, and the primary microbiome endpoint, change in relative abundance of Bifidobacterium spp. from baseline to week 12, was not met. Given the small sample size and the endpoint hierarchy, this finding should be regarded as a promising but preliminary signal rather than definitive evidence of clinical benefit.

Fecal microbiota transplantation (FMT) from healthy donors or from immunotherapy responders to immunotherapy-resistant patients is another strategy under investigation for reshaping the gut microbiota. The phase 1 PERFORM trial primarily assessed the safety of FMT plus immunotherapy (e.g., ipilimumab plus nivolumab) in 20 treatment-naive patients with metastatic RCC and primarily assessed safety (93). The reported objective response rate (ORR) was 50% and the complete response (CR) rate was 11%, corresponding to 9/18 and 2/18 evaluable patients, respectively. Grade 3 immune-related adverse events occurred in 10/20 patients (93). Although this study also suggested that gut microbiota diversity may be associated with a lower incidence of severe immune-related adverse events (93), the absence of a randomized control group limits attribution of efficacy to FMT. The randomized, double-blind, placebo-controlled phase 2 TACITO trial subsequently evaluated FMT plus pembrolizumab and axitinib in metastatic RCC (94). In this first-line study, the primary 12-month PFS comparison was 70% versus 41% (p = 0.053), which did not meet conventional statistical significance. The reported median PFS difference of 24.0 versus 9.0 months (HR 0.50, p = 0.035) was a secondary endpoint finding observed without adjustment for multiplicity. Reporting only the latter may overstate the certainty of efficacy. Overall, interventions targeting the gut microbiota are steadily moving from theory to clinical practice. Microbiome-modifying strategies, including probiotic supplementation and FMT, may improve immunotherapy outcomes in selected patient populations, particularly in first-line or treatment-naive settings. However, current evidence does not yet establish that these interventions reverse established ICI resistance. Larger randomized controlled trials with clearly defined primary endpoints, adequate control arms, and well-characterized patient populations are needed before microbiome interventions can be considered strategies to overcome ICI resistance.

## 4 Summary and future perspectives

The resistance of RCC to immunotherapy is not driven by a single mechanism but by a multi-layered network spanning tumor-intrinsic, microenvironmental, and host-related factors.

Among these, several findings genuinely change the interpretation of resistance. First, PBRM1 loss is not a uniform resistance biomarker, and its effect depends on treatment context, co-mutation status (for example, concurrent RAD51 deficiency identifies an immune-sensitive subgroup (17), and the definition of mutation versus protein loss. Second, the spatial and functional heterogeneity of the TME means that the same molecular axis can exert opposite effects in different patients. Mechanisms supported by clinical-cohort and/or functional evidence include hypoxia-HIF signaling, immunosuppressive myeloid cells, such as TAMs and MDSCs, alternative immune checkpoints, and CAF/ECM barriers. Third, host-related factors such as gut microbiome dysbiosis are supported by large real-world evidence for antibiotic exposure and early-phase randomized trial evidence for microbiome modulation (95, 96), whereas age, and the obesity paradox currently remain weaker, context-dependent stratification variables rather than established intervention targets. These distinctions shift therapeutic priorities. Interventions should focus on mechanisms with demonstrated druggability and clear direction of effect, while mechanisms that remain hypothesis-generating should be studied as stratification variables rather than treated as established resistance determinants.

First-line ICI+TKI combinations, supported by KEYNOTE-426, CheckMate 9ER, and CLEAR, delay resistance relative to sunitinib in ICI-naive patients, but they do not demonstrate reversal of established ICI resistance. In the post-progression setting, randomized evidence from CONTACT-03 and TiNivo-2 shows that ICI rechallenge does not improve PFS and may increase toxicity, and these findings should be incorporated into clinical decision-making. By contrast, later-line agents with ICI-independent mechanisms, such as the HIF-2α inhibitor belzutifan, have demonstrated PFS and response benefits over everolimus in LITESPARK-005, although OS did not differ significantly at the reported interim analysis. This represents later-line antitumor activity rather than restored ICI sensitivity. In addition, the microbiome modulator CBM588 has shown preliminary signals in first-line or treatment-naive settings, but does not yet establish reversal of established ICI resistance. Future trials should explicitly define whether they aim to prevent resistance, overcome resistance after progression, or achieve re-sensitization, and should include appropriate control arms to distinguish these objectives.

Looking ahead, the key breakthroughs in overcoming RCC immunotherapy resistance lie in establishing reliable efficacy prediction systems, analyzing the spatiotemporal heterogeneity of the TME, and optimizing sequential treatment strategies. A multidimensional prediction model integrating TMB, gene mutation profiles, immune cell subpopulation characteristics, and gut microbiome information is a promising research tool but has not yet been established as a standardized prerequisite for precision treatment. Dynamic mapping the evolutionary trajectories of different cell populations in the TME under treatment pressure may reveal new targets and timing for resistance interventions. With the widespread adoption of first-line immunotherapy combinations, clarifying the optimal subsequent therapy under different resistance mechanisms, such as VEGF-TKI monotherapy, HIF-2α inhibition, or other ICI-independent agents, remains an area of active investigation and will be essential to maximize OS benefits. Finally, because resistance-directed intensification inevitably increases toxicity, future trials should report safety and tolerability alongside efficacy endpoints and define whether the goal is prevention, overcoming, or re-sensitization, so that survival gains are not offset by treatment-related harm.
